# From Co-Administration to Co-Formulation: The Race for New Vaccines against COVID-19 and Other Respiratory Viruses

**DOI:** 10.3390/vaccines11010109

**Published:** 2023-01-02

**Authors:** Daniele Focosi

**Affiliations:** North-Western Tuscany Blood Bank, Pisa University Hospital, 56124 Pisa, Italy; daniele.focosi@gmail.com

Combined (concomitant or synchronous) vaccination is crucial to increasing the compliance rate during mass campaigns by reducing the time to deployment (i.e., the time to achieve herd immunity), congestion in healthcare facilities, and the public’s fatigue towards vaccinations (vaccine hesitancy). Eventually, the overall cost of vaccines can also be reduced. Combined vaccination can take different forms. Different vaccines (e.g., pneumococcus and influenza vaccines) can be simultaneously co-administered (typically on different body sites), or a single vaccine can be co-formulated to include antigenic components from different microbes. In the latter case, the vaccine is called polyvalent.

While antigens can come from different serotypes of a single microorganism (e.g., the bivalent COVID-19 vaccines consisting of both the wild-type and the BA.4/5 Spike proteins, or the 13-valent pneumococcal vaccines), the most common form of polyvalent vaccination consists of antigens from different microbes. Examples of polyvalent vaccines of great success include diphtheria, tetanus, and pertussis (DTP), orinactivated polio, *Haemophilus influenzae*, and hepatitis B vaccines. However, the development of such polyvalent formulations has encountered numerous challenges, including reduced immunogenicity (e.g., *Hemophilus influenzae*) or worsened safety (e.g., DTP).

Worldwide, influenza infection leads to 3–5 million severe cases and 290,000–650,000 influenza-related respiratory deaths annually, despite the availability of current influenza vaccines. Although both influenza A and B cause seasonal epidemics, it is the influenza A that lead to >95% of hospitalization in adults. In the US, influenza causes 140,000 to 710,000 hospitalizations, 12,000 to 52,000 deaths, and about USD 25 billion in economic losses annually [1]. In 2022, the co-administration of COVID-19 vaccines with influenza vaccines [2,3,4,5] and/or pneumococcal vaccines [6] was deployed, with safety fully demonstrated in spite of minimal concerns regarding immunogenicity [7]. With the decreasing compliance rates for COVID-19 boosters, co-formulations are becoming increasingly appealing.

As both the influenza virus and SARS-CoV-2 continue to circulate and evolve, the combination of these vaccines into a single shot could offer protection against both diseases. Unlike the influenza virus, SARS-CoV-2 is not yet subject to antigenic shifts; however, thus far, the degree of antigenic drift has been enough to justify the upgrading of vaccines [8]. Even though COVID-19 has not shown the seasonal pattern observed for influenza, its main target groups are largely the same (i.e., frail elderlies), and, so far, the mean duration of COVID-19 vaccines’ efficacy against severe disease is around one year, leaving room for synchronous administration of COVD-19 and seasonal flu boosters. Consequently, vaccine manufacturers have been increasingly investigating co-formulated influenza and COVID-19 vaccines.

From a commercial standpoint, co-formulation requires each manufacturer to develop ingredients for both vaccines. Novavax completed a phase I/II trial of its trivalent NanoFlu^®^ candidate vaccine (wild-type recombinant influenzavirus hemagglutinin (rHA) proteins (qNIV2) in Matrix-M1™ adjuvant) in October 2018 (NCT03293498), the phase 2 trial of the quadrivalent candidate on 1375 participants in April 2019 (NCT03658629), and the phase 3 randomized trial of the quadrivalent vaccine against Fluzone^®^ on 2650 adults in November 2020 (NCT04120194). However, none of the two main COVID-19 mRNA vaccine manufacturers (Moderna and Pfizer/BioNTech) had ever marketed a flu vaccine. Notably, mRNA technology has the potential to shorten manufacturing times, thereby postponing the forecast of the dominant influenza lineages, and hence improving prediction accuracy.

Following successful phase 1/2 studies (NCT05606965 and NCT04956575), Moderna initiated two phase 3 trials of its mRNA flu vaccine, mRNA-1010, among a population of 6102 adults in the Southern hemisphere on 7 June 2022 (NCT05415462) and among 23,000 adults in the Northern hemisphere on 14 September 2022 (NCT05566639). Following successful phase 1/2 study (NCT05052697) in 1980, since September 2022 Pfizer is investigating its quadrivalent modRNA-based influenza vaccine candidate, qIRV (which incorporates four different influenza strains and is recommended by the WHO for use in the Northern Hemisphere by 2022/23) in a randomized controlled trial (NCT05540522) using a population of 25,000 US subjects [9].

On 3 November 2022, Pfizer and BioNTech announced a phase 1 trial of a combined COVID-19 and influenza vaccine that will enroll 180 American adults aged 18–64 (NCT05596734) and is expected to be completed by June 2024 [10]. This vaccine candidate combines Pfizer’s quadrivalent qIRV and Pfizer and BioNTech’s authorized Omicron-adapted bivalent COVID-19 BNT162b2 (wild-type/Omicron BA.4/BA.5) vaccine.

The Novavax COVID-19–NanoFlu combination investigational vaccine uses the full-length, stabilized, recombinant SARS-CoV-2Spike protein (NVX-CoV2373) and NanoFlu as antigens organized into distinct nanoparticle complexes [11]. Tested in ferrets and hamsters, the shot produced antibodies against both viruses at levels comparable to those offered with either constituent vaccine alone and protected the animals from a SARS-CoV-2 challenge [12]. A phase I/II trial at 12 sites in Australia incorporating 642 healthy adults aged 50–70 years (NCT04961541) was completed in April 2022. A phase 2 trial incorporating 2300 adults aged 50–80 (NCT05519839) will be launched in January 2023, which is expected to reach completion by July 2023.

Moderna is testing its mRNA-1073 COVID-19/influenza mRNA vaccine in a phase 1/2 trial on 550 adults, which should be completed by June 2023 (NCT05375838); meanwhile, it is also investigating another cocktail of two (mRNA-1045: influenza and RSV) and a cocktail of three (mRNA-1230: Influenza, RSV, and SARS-CoV-2) vaccines in 675 adults aged 50 to 75 years, which is expected to completed by November 2023 (NCT05585632). This product will likely follow the soon-to-be-available mRNA-1345 monovalent RSV vaccine. *Streptococcus pneumoniae*, whose co-infection with COVID-19 leads to lethal synergy in animal models, is another promising candidate for co-formulation [13].

More manufacturers can join this race, as many different co-formulated vaccines are still under preclinical development [14,15,16,17,18], including live-attenuated influenza virus (LAIV) vector-based vaccines [19,20,21]. Among the latter, the intranasal candidate developed by Beijing Wantai Biological Pharmacy Enterprise achieved poor immunogenicity in a phase 1/2 clinical trial [22].

While the public’s compliance to COVID-19 vaccines is declining, the increased circulation of both RSV and influenza virus in 2022 [23] has attracted public attention. Attitudes towards a hypothetical co-formulated COVID-19/influenza vaccine were estimated at 50% in a recent and large-scale (n = 12,887) United States survey [24].

Recently, a universal mRNA vaccine against all 20 known influenza virus subtypes was validated in animal models [25], and this may represent an ideal component for a pan-respiratory vaccine. A pan-respiratory vaccine is likely the asymptote to which each manufacturer will tend, but competition, patents, and market litigations will likely make this goal very difficult to achieve.
